# Supplementary material for: Causal Relationship between Aging and Anorexia Nervosa: A White-Matter-Microstructure-Mediated Mendelian Randomization Analysis
Source: Biomedicines. 2024 Aug 16;12(8):1874. doi: 10.3390/biomedicines12081874 (PMC11351342; doi:10.3390/biomedicines12081874)
Supplement: Supplementary file 1 [file biomedicines-12-01874-s001.zip › Supplementary Figure/Figure S4/Singe SNP plot of MR analysis for TL to ubm-b-1642.pdf]

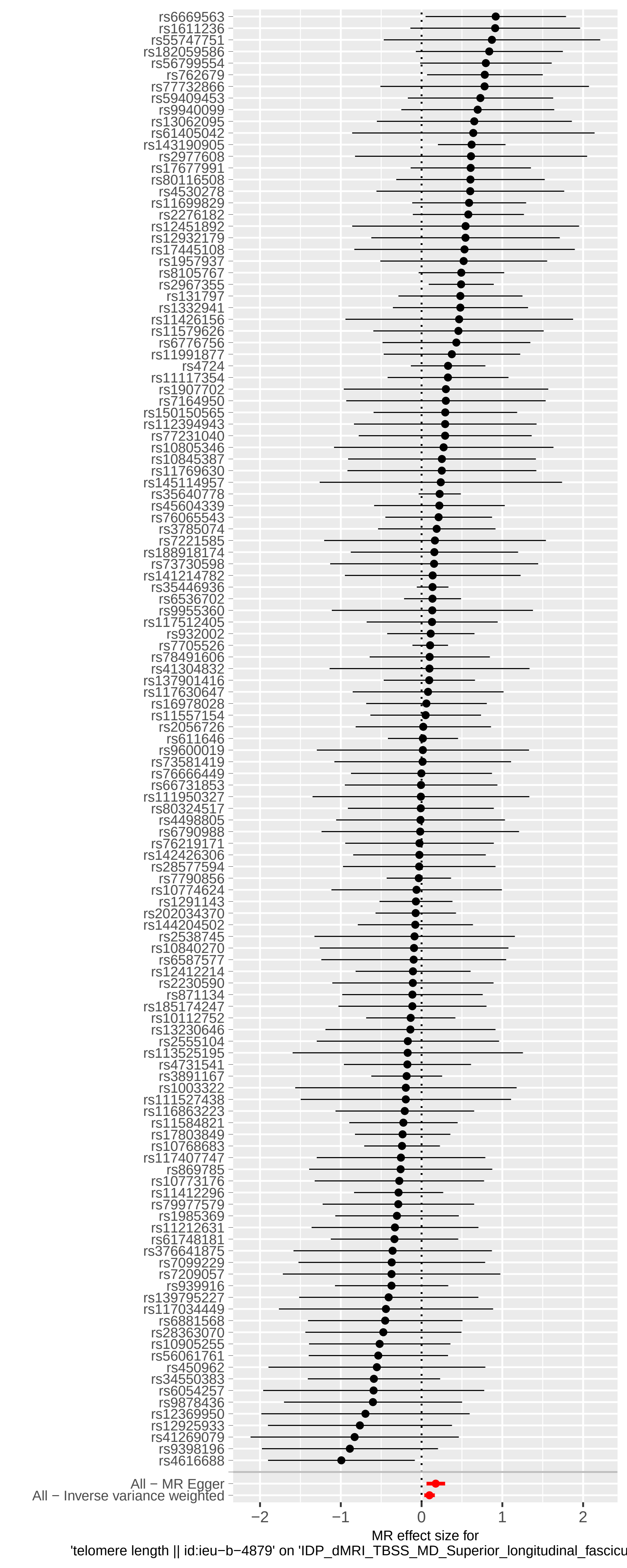
rs6669563
rs1611236
rs55747751
rs182059586
rs56799554
rs762679
rs77732866
rs59409453
rs9940099
rs13062095
rs61405042
rs143190905
rs2977608
rs17677991
rs80116508
rs4530278
rs11699829
rs2276182
rs12451892
rs12932179
rs17445108
rs1957937
rs8105767
rs2967355
rs131797
rs1332941
rs11426156
rs11579626
rs6776756
rs11991877
rs4724
rs11117354
rs1907702
rs7164950
rs150150565
rs112394943
rs77231040
rs10805346
rs10845387
rs11769630
rs145114957
rs35640778
rs45604339
rs76065543
rs3785074
rs7221585
rs188918174
rs73730598
rs141214782
rs35446936
rs6536702
rs9955360
rs117512405
rs932002
rs7705526
rs78491606
rs41304832
rs137901416
rs117630647
rs16978028
rs11557154
rs2056726
rs611646
rs9600019
rs73581419
rs76666449
rs66731853
rs111950327
rs80324517
rs4498805
rs6790988
rs76219171
rs142426306
rs28577594
rs7790856
rs10774624
rs1291143
rs202034370
rs144204502
rs2538745
rs10840270
rs6587577
rs12412214
rs2230590
rs871134
rs185174247
rs10112752
rs13230646
rs2555104
rs113525195
rs4731541
rs3891167
rs1003322
rs111527438
rs116863223
rs11584821
rs17803849
rs10768683
rs117407747
rs869785
rs10773176
rs11412296
rs79977579
rs1985369
rs11212631
rs61748181
rs376641875
rs7099229
rs7209057
rs939916
rs139795227
rs117034449
rs6881568
rs28363070
rs10905255
rs56061761
rs450962
rs34550383
rs6054257
rs9878436
rs12369950
rs12925933
rs41269079
rs9398196
rs4616688
All − MR Egger
All − Inverse variance weighted
−2
−1
0
1
2
MR effect size for
'telomere length || id:ieu−b−4879' on 'IDP_dMRI_TBSS_MD_Superior_longitudinal_fascicu
